# Editorial: Chemical-genetics to study microbial pathogen basis of infection and drug targets

**DOI:** 10.3389/fcimb.2025.1582879

**Published:** 2025-03-19

**Authors:** Aditya S. Paul

**Affiliations:** Department of Immunology and Infectious Diseases, School of Public Health, Harvard University, Boston, MA, United States

**Keywords:** pathogen, Plasmodium, Babesia, Cryptosporidium, Candida, Fasciola, *Galleria mellonella*

Infection microbiology is challenging because pathogens develop adaptations that elude straightforward analogy with non-pathogens. Experimental methods for pathogens typically lag behind model systems, with few “off-the-shelf” approaches for direct study. Nonetheless, dedicated activity by smaller communities of researchers has over time yielded tools for study that are proving powerful for detailed understanding of infection processes. Reverse genetic approaches and chemical probe inhibitors are providing insights into pathogenic mechanisms. Identification of chemical susceptibilities of microbial pathogens has clear relevance for therapeutic interventions and drug development.

We have gathered 5 articles that utilize chemical biology and reverse genetic approaches to study infectious fungi, parasitic protists, and parasitic worms. The through line across the studies is the use of inhibitors in assays of infection, allowing researchers to study the basis of chemical susceptibility in poorly understood pathogenic organisms. Readers will appreciate the specialized approaches required to sustain these systems in the lab and make them amenable to measurements of growth and infection in controlled environments.

The phylum Apicomplexa comprise a diverse group of parasites of widespread medical and veterinary importance. Despite the longstanding need for interventions, drugs and treatments are few. Cryptosporidiosis is a diarrheal disease, for which there exists one FDA-approved chemotherapy (Sparks et al., 2015). Over the past decade, methods for continuous propagation of *Cryptosporidium* parasites in a lab setting have come online (Vinayak et al., 2015), enabling experimental approaches to study biology and chemical susceptibility. Here, Hanna et al. carry out an elegant set of chemical-genetic studies to prove a target of a potent antiparasitic inhibitor in *Cryptosporidium parvum*, one of the species that cause human disease. Following chemoproteomics, the researchers use knockdown, overexpression, and site-directed mutagenesis, to demonstrate specific targeting of a tRNA-synthetase in *C. parvum*. This study, with an earlier report from another group (Vinayak et al., 2020), expanded the set of selectable markers in *C. parvum* to include resistant alleles of parasite tRNA-synthetases. Also within Apicomplexa, the sister genera *Plasmodium* and *Babesia* are home to parasite species with specific tropism for red blood cells; in humans these parasites cause malaria and babesiosis, respectively. While treatments have tended to focus on molecular targets within the parasites, Groomes et al. undertook a screen of *Plasmodium* and *Babesia* parasites with drugs and advanced clinical-stage inhibitors annotated for targets in the host red blood cell. The researchers identified micromolar-IC50 potency inhibitors amongst these compounds, with higher potency generally displayed toward the *Plasmodium* parasites. In addition to the potential for repurposing, the study utilized assays that provide direct experimental for host cell targeting required for antiproliferative action.

*Candida auris* is a recently emerged fungal pathogen present in healthcare settings (Lockhart et al., 2017), with drug-resistance being a major issue (Du et al., 2020). Following a chemical screen in *C. auris* that identified an aryl-carbohydrazide inhibitor of proliferation, Tebbji et al. carry out a chemical-genetic haploinsufficiency profiling screen in the better established laboratory model *Candida albicans*. The researchers show that *C. albicans* cells with reduced expression of the fatty acid desaturase Ole1 gene exhibit increased susceptibility to the inhibitor. Subsequent fatty acid metabolism studies as well as supplementation experiments supported the Ole1-pathway as a target of the inhibitor in *C. albicans* and *C. auris*. In a moth larva model (*Galleria mellonella*) of systemic candidiasis caused by infection with *C. auris* or *C. albicans*, administration of the original hit compound or an analog improved survival.

We included in this Research Topic a study on parasitic *Fasciola* flukes, the causative agent of the zoonotic disease fascioliasis. Though not a microorganism (the worms can grow up to several centimeters in length), the use of chemical inhibitors to study trematode parasite development throughout the course of host infection aligns well with the research theme. Juvenile worms mature into adults in their journey from the stomach towards the liver, as the surroundings become increasingly anaerobic. Based on analogy to nematodes (Iwata et al., 2008), Tashibu et al. use a combination of biochemistry and chemical biology to find that juvenile and adult worms differ in their modes of mitochondrial respiration. The researchers found that juveniles carry out conventional ubiquinone-dependent respiration in the presence of oxygen, but can also perform a non-canonical, rhodoquinone-dependent mode of fumarate respiration to produce ATP. Adults, by contrast, predominantly utilize the fumarate mode of respiration. The chemical biology approach in this study was critical in demonstrating changes in mitochondrial metabolism during worm maturation, tracking with changes in oxygen within developmental niches in the host organism.

A translational framework also touches on improved pipelines for screening antimicrobial therapies. Guarnieri et al., in their studies of lactic acid bacteria (LAB) as a topical anti-infective, introduce the use of *G. mellonella* as an alternative to vertebrate animals for facile screening of LAB for burn wounds.

Chemical biology is a growing area for study of pathogens and infection biology. In some systems, the availability and implementation of genetics has allowed researchers to study the modes of action of compounds in live microbes, a powerful adjunct to traditional biochemical approaches. The implications are significant, especially given that frequently the related diseases have few treatments. Identification of a target can shed light on infection mechanisms and may inform new drug development efforts.
